# Mitochondrial Genome of *Scutiger ningshanensis* (Anura, Megophryidae, *Scutiger*): Insights into the Characteristics of the Mitogenome and the Phylogenetic Relationships of Megophryidae Species

**DOI:** 10.3390/genes16080879

**Published:** 2025-07-26

**Authors:** Siqi Shan, Simin Chen, Chengmin Li, Lingyu Peng, Dongmei Zhao, Yaqing Liao, Peng Liu, Lichun Jiang

**Affiliations:** Key Laboratory for Molecular Biology and Biopharmaceutics, School of Biological and Pharmaceutical Sciences, Mianyang Teachers’ College, Mianyang 621000, China; 19839412383@163.com (S.S.); 17709049484@163.com (S.C.); 18380527400@163.com (C.L.); 15984106856@163.com (L.P.); 17808309011@163.com (D.Z.); 17873081786@163.com (Y.L.); liupeng2023@mtc.edu.cn (P.L.)

**Keywords:** *Scutiger ningshanensis*, mitochondrial genome, phylogenetic analysis, divergence time, Ka/Ks

## Abstract

**Background/Objectives:** *Scutiger ningshanensis* (Fang, 1985) is an endemic Chinese amphibian species within the genus *Scutiger* (Megophryidae). Despite its ecological significance, its mitochondrial genome architecture and evolutionary relationships remain poorly understood. Given the high structural variability in Megophryidae mitogenomes and unresolved phylogenetic patterns in *Scutiger*, this study aims to (1) characterize the complete mitogenome of *S. ningshanensis*, (2) analyze its molecular evolution, and (3) clarify its phylogenetic position and divergence history within Megophryidae. **Methods:** The complete mitochondrial genome was sequenced and annotated, followed by analyses of nucleotide composition, codon usage bias, and selection pressures (Ka/Ks ratios). Secondary structures of rRNAs and tRNAs were predicted, and phylogenetic relationships were reconstructed using maximum likelihood and Bayesian methods. Divergence times were estimated using molecular clock analysis. **Results:** The mitogenome of *S. ningshanensis* is 17,282 bp long, encoding 13 protein-coding genes (PCGs), 22 tRNAs, 2 rRNAs, and a control region, with a notable AT bias (61.05%) with nucleotide compositions of T (32.51%), C (24.64%), G (14.3%), and A (28.54%). All tRNAs exhibited cloverleaf structures except trnS1, which lacked a DHU stem. Phylogenetic analysis confirmed the monophyly of *Scutiger*, forming a sister clade to *Oreolalax* and *Leptobrachium*, and that *S. ningshanensis* and *S. liubanensis* are sister species with a close evolutionary relationship. Positive selection was detected in Atp8 (Ka/Ks > 1), suggesting adaptation to plateau environments, while other PCGs underwent purifying selection (Ka/Ks < 1). Divergence time estimation placed the origin of Megophryidae at~47.97 MYA (Eocene), with *S. ningshanensis* diverging~32.67 MYA (Oligocene). **Conclusions:** This study provides the first comprehensive mitogenomic characterization of *S. ningshanensis*, revealing its evolutionary adaptations and phylogenetic placement. The findings enhance our understanding of Megophryidae’s diversification and offer a genomic foundation for future taxonomic and conservation studies.

## 1. Introduction

The systematic classification and evolutionary relationships of anurans (Anura), one of the most diverse and widely distributed amphibian groups, remain a key focus in evolutionary biology [1]. Among these, the Megophryidae family (Bonaparte, 1850) represents an endemic Asian taxon predominantly distributed across the Himalayas and adjacent regions, including northern Myanmar, Nepal, and southwestern China [2]. Molecular phylogenetic studies have consistently supported the validity and monophyly of Megophryidae [3,4], with *Scutiger* (Theobald, 1868) recognized as a distinct lineage sister to *Oreolalax* within this family [5].

*S. ningshanensis* is a rare amphibian endemic to China, classified as a national second-grade protected species. It primarily inhabits high-elevation mountain streams in Shaanxi, Henan, and surrounding regions [6]. Molecular barcoding studies initially identified populations in the East Qinling Mountains as a cryptic species within the *S. ningshanensis* group [7], a conclusion later confirmed through phylogenetic and morphological analyses.

Recent advances in next-generation sequencing have spurred increased research into the evolutionary history and interspecific relationships of *Scutiger* species [8]. While mitochondrial gene fragments (e.g., *12S rRNA*, *16S rRNA*, *Cytb*) and nuclear markers (e.g., *RAG1*, *c-myc2*, *ccn-B3*, *b-fib7*) have been employed to reconstruct phylogenies, these studies have revealed complex patterns, including hybridization among species such as *S. boulengeri*, *S. glandulatus*, *S. tuberculatus*, and *S. mammatus* [9,10]. Further analyses incorporating additional taxa (*S. liupanensis*, *S. sikimmensis*, *S. occidentalis*, *S. nepalensis*) suggest a Paleo-Tibetan origin for the genus, with evidence of cryptic diversity [11].

Despite these advances, the lack of complete mitochondrial genome data for *Scutiger* limits comprehensive assessments of genetic diversity and phylogenetic resolution. Mitochondrial genomes, due to their maternal inheritance, moderate evolutionary rate, and structural conservation, are invaluable for population genetics, phylogenetics, and divergence time estimation [12,13,14]. Their higher mutation rate compared to nuclear DNA makes them particularly useful for resolving recent speciation events and population structures [15,16,17,18,19].

This study aims to sequence and analyze the mitochondrial genome of *S. ningshanensis*, examining its gene composition, base content, codon usage, and selection pressure to infer phylogenetic relationships and divergence times. The findings will enhance our understanding of Megophryidae evolution and provide a foundation for future taxonomic, phylogenetic, and conservation studies within this family.

## 2. Materials and Methods

### 2.1. Specimen Collection and DNA Extraction

This experiment was conducted using *S. ningshanensis* specimens from Pingheliang Mountains, Ningshan County, Shaanxi Province (N 39°33′37.18″, E 94°53′14.57″, 2157 m above sea level). In this study, we used a combination of morphology and molecular biology for species identification. First, preliminary identification was made through the information of the collection site and the morphological characteristics of the specimen (body length of 46 mm, body color of bright brown, and flat, narrow, and long body shape, etc.). Subsequently, based on the identifying characteristics of *S. ningshanensis* in FRPS, we focused on the body shape, skin structure (some small warts on the whole dorsal surface between the skin folds or large warts, etc.), morphology of the fingers and toes (the webbing of the toes is undeveloped, etc.), as well as the secondary sexual characteristics (black thorns on the two sides of the head, including the labial margins, the eyelids, the temporal folds, etc.), and determined that this specimen was *S. ningshanensis*.

All procedures involving *S. ningshanensis* specimens were conducted in strict compliance with the Wildlife Protection Law of the People’s Republic of China. For genetic sampling, we employed a non-lethal protocol: the interdigital webbing was first sterilized with alcohol, approximately 30 mg of tissue was carefully excised, and the area was disinfected again before releasing the animal back into its natural habitat. Immediately following collection, specimens were preserved under aseptic conditions in anhydrous ethanol to maintain tissue integrity. For DNA extraction, we followed the manufacturer’s protocol of a commercial DNA extraction kit, using muscle tissue from the flippers as the source material. The quality of extracted DNA was initially verified through 0.8% agarose gel electrophoresis. Subsequently, DNA concentration and purity were precisely quantified using spectrophotometric analysis. All processed DNA samples were aliquoted and stored at −20 °C to ensure long-term stability for future molecular analyses. Amplify *COI* gene and compare it in NCBI database to further confirm that the collected sample was *S. ningshanensis*.

### 2.2. Primer Design and PCR Amplification

Using primer design software, specific primers covering the entire mitochondrial genome were created based on known mitochondrial genome sequences from the same genus and related species. To amplify the target mitochondrial DNA regions, PCR primers were designed using a dual approach: (1) alignment-based selection targeting conserved genomic regions, and (2) adaptation of published primers from Kurabayashi and Sumida [20]. A total of 12 primer pairs (Table 1) were subsequently employed for PCR amplification under optimized reaction conditions. Polymerase chain reaction (PCR) technology was used to amplify the target regions. Each 50 μL PCR reaction contained 5× PrimeSTAR GXL Buffer, dNTP mixture, PrimeSTAR GXL DNA Polymerase (TaKaRa), 1 μM forward and reverse primers, and an appropriate amount of template DNA. The PCR cycle conditions were as follows: 3 min of pre-denaturation at 95 °C, 30 s of denaturation at 94 °C, 30 s of annealing at 52–62 °C, 30 cycles of extension at 72 °C for 90 s, 10 min of final extension at 72 °C, and storage at 4 °C. All purified amplification products were subjected to direct automated DNA sequencing using the Sanger method after identification by agarose gel electrophoresis to confirm their size.

### 2.3. Sequence Assembly, Analysis, and Annotation

The purified PCR amplicons were subjected to bidirectional sequencing employing the Sanger method (3730xl Genetic Analyzer, Applied Biosystems, Waltham, MA, USA). Subsequent sequence assembly was performed using DNA Baser software (version 5.20; http://www.DNABaser.com, accessed on 20 May 2025.), utilizing 200–350 bp overlapping regions between contigs to reconstruct the complete mitochondrial genome with high fidelity. Using MEGA11.0 [21] software, the relative synonymous codon usage (RSCU values), genetic differences between species, individual base composition occupancies, and A + T content were calculated to translate all 13 PCGs into their corresponding amino acid sequences. The online tool called MITOS was used to annotate the mitochondrial genome [22]. Mitochondrial genome mapping was constructed using OGDRAW and default parameters [23]. We used tRNAscan-SE and the Mitos Web server [24] in default search mode to analyze tRNA secondary structures using vertebrate mitochondrial genetic code sources. R2DT is an online tool used to predict the tRNA structure. The formulas for AT-bias = (A −T)/(A + T) and GC-bias = (G − C)/(G + C) [25] were used to calculate nucleotide composition bias, and manual calculations were performed to determine gene overlap and intergenic spacer regions. Comparing homologous sequences of related species allowed for the identification of the conserved start regions of light chain duplication (*O_L_*) and the control region (*D-loop*, CR).

### 2.4. Phylogenetic Analyses

To investigate the systematic position of *S. ningshanensis* within its family, we constructed a phylogenetic tree using 28 representative species of 5 families (Megophryidae, Pelobatidae, Pelodytidae, Leiopelmatidae, and Bombinatoridae), along with 2 species from different families as outgroups. Phylogenetic analysis of tandem sequences of 13 PCGs from the entire mitotic genome was performed using PhyloSuite_v1.2.3 [26], and before constructing the phylogenetic tree, all the sequences were aligned using MAFFT (version 7) [27]. Then the sequences were cut using the Gblocks tool, and the most suitable data model was determined according to the AIC (Akaike Information Criterion) [28] criteria, and finally the phylogenetic tree was constructed using Maximum Likelihood (ML) and Bayesian Inference [29] (BI). Inferred phylogenies were inferred using MrBayes 3.2.6 inferred Bayesian phylogenies under a partitioned model (2,000,000 generations with a sampling frequency of 1000) in which the initial 25% of the sampled data were discarded as aged data. The maximum likelihood phylogeny was inferred using IQ-TREE [30]. Branch support values for Bayesian posterior probabilities and maximum likelihood Bootstrap support values were displayed on the tree. Ultimately, the evolutionary relationship between *S. ningshanensis* and the family Megophryidae was resolved by assessing support for different tree forms.

### 2.5. Divergence Time Estimates

The BEAST program was utilized to estimate divergence times based on the 13 protein-coding genes (PCGs) from each species [31]. Divergence times were estimated using rigorous molecular clocks and GTR evolutionary models. Tree priorities were determined through Yule processes. We calibrated the divergence times using fossil records from the TimeTree website. The following divergence times were established as fossil calibration points: 18.5–48.7 million years ago (MYA) for the genera *Scutiger* and *Oreolalax*, 20.0–57.5 MYA for the genus *Leptobrachium*, and 33.3–66.3 MYA for the genus *Leptobrachella*. After 20 billion generations, the first 10% of the MCMC (Markov Chain Monte Carlo) chain was discarded to account for aging effects. Effective sample size (ESS) values were confirmed using Tracer [32], and parameters were recorded every 2000 generations, with all ESS values exceeding 200. The maximum clade credibility (MCC) tree was visualized and edited using FigTree version 1.4.5, while TreeAnnotator version 1.8.4 was employed to generate the tree.

### 2.6. Ka and Ks Analysis

Selection pressure acting on *S. ningshanensis* was assessed using the Ka/Ks ratio. This metric compares the rate of non-synonymous substitutions per non-synonymous site (Ka) to the rate of synonymous substitutions per synonymous site (Ks). Ka/Ks values were calculated between 13 protein-coding genes using KaKs_Calculator 2.0 (YN model). CDS sequences were filtered for low-quality sites by MAFFT [27] comparison and Pal2Nal codon comparison. Positively selected genes were required to fulfill the following criteria: (1) Ka/Ks > 1 (*p* < 0.05), (2) Bonferroni correction was significant. A Ka/Ks value greater than 1 indicates positive selection, while a value equal to 1 suggests neutral selection. If the Ka/Ks value is less than 1, it indicates the presence of purifying selection. This method allows for a more in-depth examination of evolutionary trends and highlights the different selective forces that influence the genetic makeup of various species [33].

## 3. Results

### 3.1. Characterization of the Mitogenome Structure

**Figure 1:**
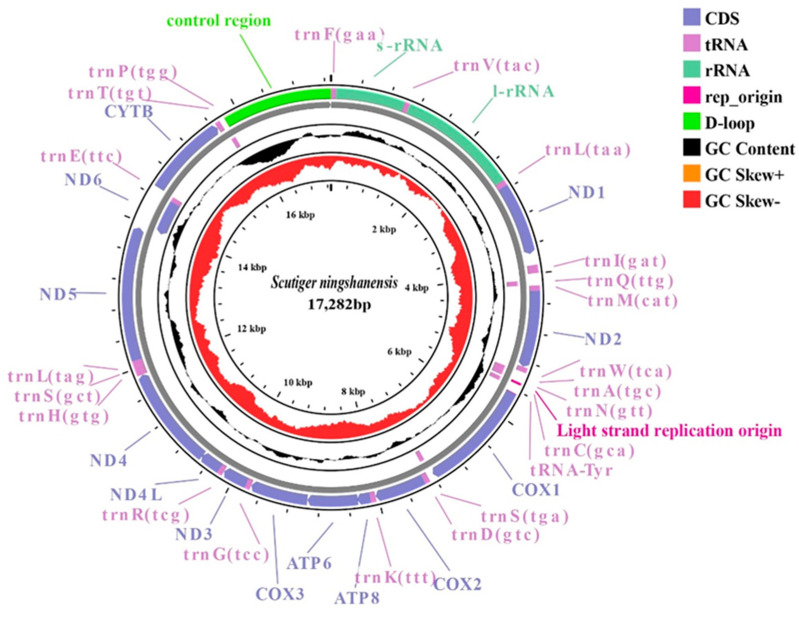
Complete mitogenome circles map of *S. ningshanensis*. Purple represents tRNA, green represents rRNA, black areas represent GC content; in addition, the map also shows the GC-skew value.

The complete mitochondrial genome (mitogenome) of *S. ningshanensis* (GenBank: PV083741) is a circular molecule of 17,282 bp, containing 13 protein-coding genes (PCGs), 2 rRNAs (*12S rRNA* and *16S rRNA*), 22 tRNAs, and a control region (*D-loop*) (Figure 1, Table 2). Most genes (12 PCGs, 14 tRNAs, and both rRNAs) are encoded on the heavy strand (H-strand), while only ND6 and 8 tRNAs reside on the light strand (L-strand). Five gene overlaps were identified, with the longest (10 bp) occurring between ATP6 and ATP8. The mitogenome displays a strong AT bias (61.05%), with individual nucleotide frequencies of A = 28.54%, T = 32.51%, G = 14.30%, and C = 24.64% (Table 3). Negative AT and GC skew values indicate strand-specific compositional asymmetry. Additionally, the *D-loop* region—enriched with tandem repeats and indel polymorphisms—contributes significantly to interspecies length variation, likely due to replication-associated insertions/deletions, reflecting the evolutionary plasticity of mitochondrial genomes.

### 3.2. Protein-Coding Genes and Codon Usage

Comparative analysis of the 13 protein-coding genes (PCGs) revealed conserved nucleotide composition patterns across Megophryidae species. In *S. ningshanensis*, the AT content of PCGs ranged from 57.25% to 68.33%, consistent with the strong AT bias characteristic of the family (Table 3). This genomic signature was particularly evident in the *D-loop* region, which exhibited the highest AT content, followed by PCGs, rRNAs, and tRNAs. Gene length variation was observed among PCGs, with *ND5* (1821 bp) being the longest and *ATP8* (180 bp) the shortest. Codon usage analysis showed GTG start codons in *COX1* and *ATP8*, while the remaining PCGs initiated with ATG. Three types of stop codons were identified: complete TAA (*ND1*, *ND2*, *ATP8*, *ND4L*), complete TAG (*ND5*), and AGG (*ND6*), with the remaining genes possessing incomplete T-stop codons. Strand composition analysis revealed negative AT skew in PCGs and *D-loop*, contrasting with positive AT skew in tRNAs and rRNAs. The GC skew was strongly C-biased in the complete mitogenome (−0.265) and *D-loop* (−0.349), while tRNAs showed positive GC skew and other elements displayed negative skew (Table 3). These compositional biases, particularly in AT-rich regions, likely contribute to mitogenome length variation and evolutionary dynamics in this lineage [34].

**Figure 2:**
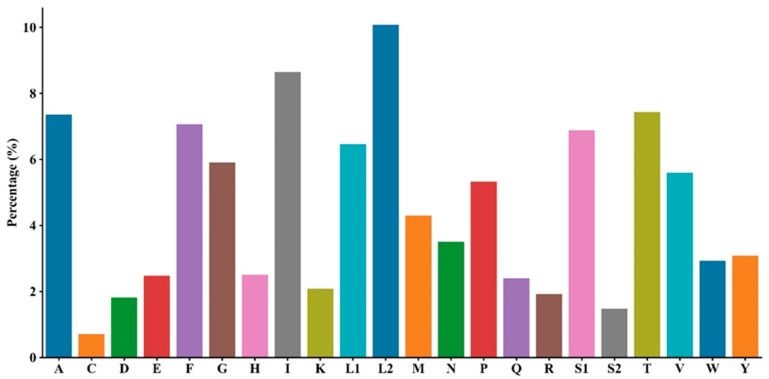
Amino acid content of PCGs in *S. ningshanensis*.

**Figure 3:**
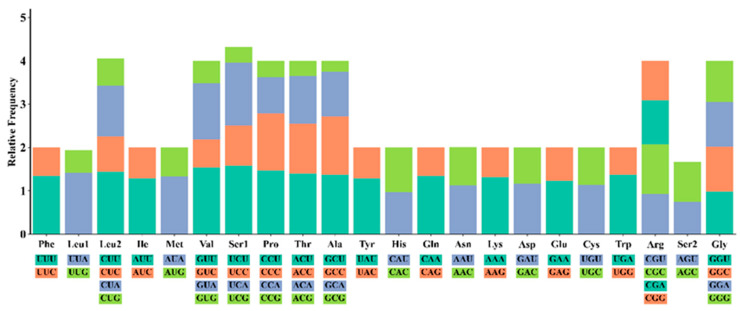
Relative synonymous codon usage (RSCU) in the mitogenome of *S. ningshanensis*. (The codon is represented by the X-axis, and the RSCU value is represented by the Y-axis).

Analysis of the 13 protein-coding genes in *S. ningshanensis* identified 5760 codons with distinct usage patterns. Leucine (Leu, 16.5%) was the most abundant amino acid, followed by isoleucine (Ile, 8.7%) and serine (Ser, 8.4%), while aspartic acid (Asp, 1.8%) and cysteine (Cys, 0.7%) were least frequent (Figure 2). Relative synonymous codon usage (RSCU) analysis revealed UCU (Ser), GUU (Val), and CCU (Pro) as the most frequent codons, whereas GCG (Ala), ACG (Thr), and UCG (Ser) were the rarest (Figure 3). Notable codon preferences were observed, including a predominance of CUU in Leu2 and UUC in Phe codons. Comparative analysis with *Oreolalax* and *Leptobrachium* species revealed subtle but significant interspecific differences in synonymous codon usage patterns. Furthermore, examination of the *ATP6* gene identified unique amino acid variations that may represent species-specific genetic signatures. These findings collectively demonstrate substantial codon usage bias in the *S. ningshanensis* mitochondrial genome, reflecting potential evolutionary adaptations.

### 3.3. Transfer RNA and Ribosomal RNA Genes and Control Region

**Figure 4:**
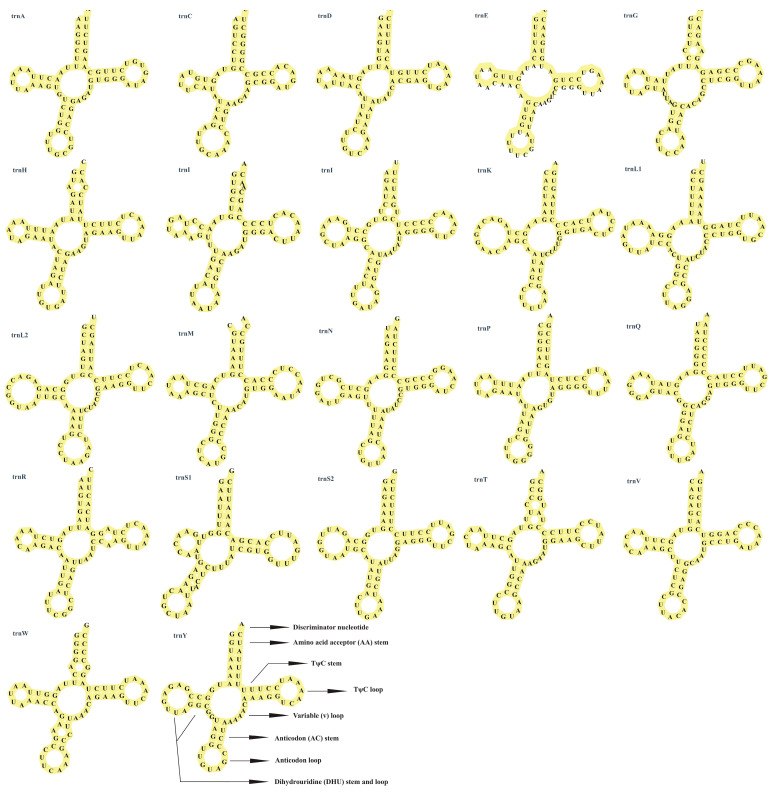
Putative secondary structure of transfer RNA (tRNA) genes of mitogenome of *S. ningshanensis*.

The mitochondrial genome of *S. ningshanensis* harbors 22 transfer RNA (tRNA) genes, collectively spanning 1504 bp, accounting for 8.7% of the entire mitochondrial genome (Table 2). All tRNAs exhibited lengths ranging from 63 bp (*tRNA-Cys*) to 75 bp (*tRNA-Leu*), consistent with typical metazoan mitochondrial tRNA sizes [35], with no intergenic overlaps observed. Secondary structure predictions using tRNAscan-SE revealed that 21 tRNAs adopt the canonical cloverleaf structure (Figure 4). The exception was *trnS1*, which lacked the dihydrouracil (DHU) stem, a structural modification commonly observed in mitochondrial genomes. The tRNA secondary structure analysis in *S. ningshanensis* revealed the presence of both standard Watson–Crick base pairs (AU and CG) and several non-canonical pairings (GU, AG, UU, AA, AC, CU, and CC) distributed throughout the stem regions. Notably, while the DHU and TΨC loops exhibited structural variability, the anticodon loop maintained high conservation (Figure 4). Quantitative assessment showed distinct mismatch frequencies with 29 GU, 2 UU, 1 CU, 4 AC, 1 AG, 1 CC, and 1 AA pairings, suggesting these non-standard pairings may represent species-specific structural adaptations that contribute to tRNA functionality and evolutionary diversification.

**Figure 5:**
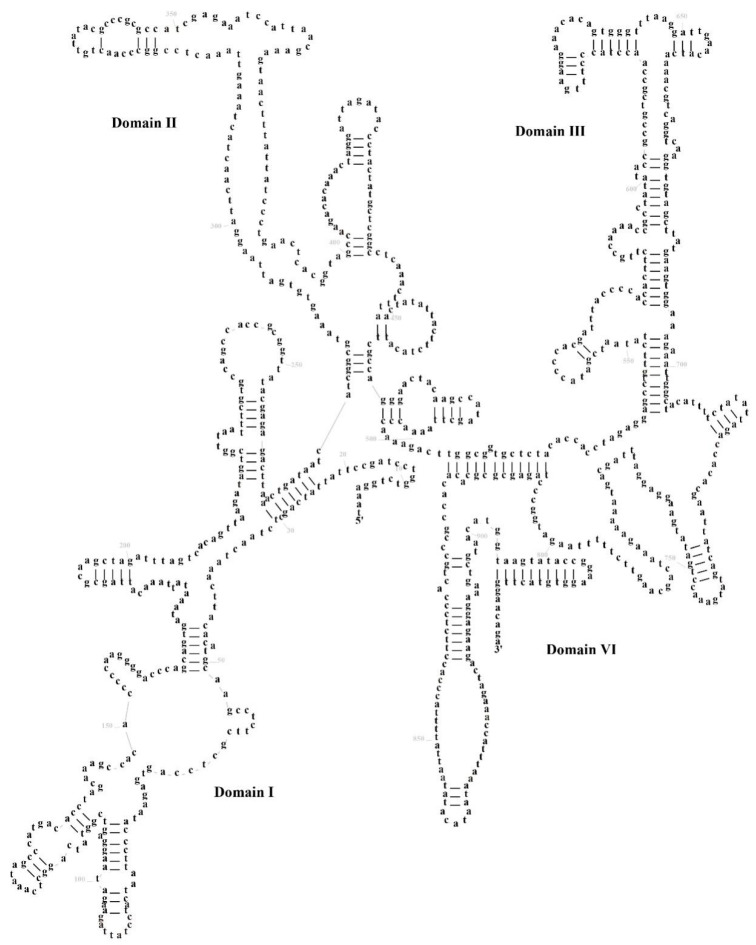
The prognostic map of *12S rRNA* secondary structures in *S. ningshanensis*.

**Figure 6:**
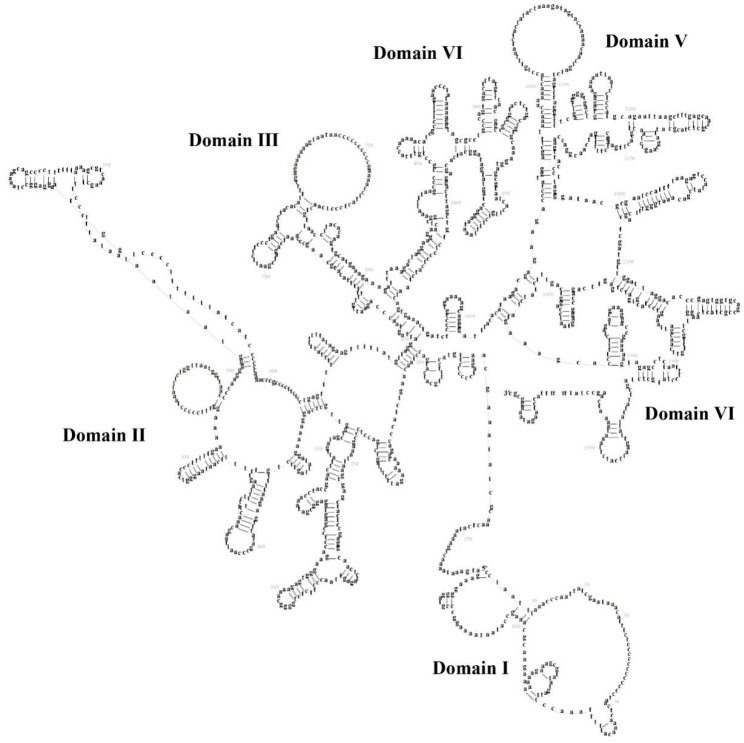
The prognostic map of *16S rRNA* secondary structures in *S. ningshanensis*.

The mitochondrial genome of *S. ningshanensis* encodes two ribosomal RNA genes: *12S rRNA* (935 bp) and *16S rRNA* (1610 bp), located between *tRNA^Phe^-tRNA^Val^* and *tRNA^Val^-tRNA^Leu^*, respectively. Secondary structure prediction revealed that *12S rRNA* comprises 4 structural domains with 43 stem-loop structures, where domains I and II represent variable regions, while III and IV are conserved (Figure 5). In contrast, *16S rRNA* exhibits a more complex architecture, consisting of 6 structural domains with 55 stem-loop structures, where domains I–IV are variable, and IV–V are conserved (Figure 6). Comparative analysis demonstrated that stem regions are evolutionarily more conserved than loop regions, and *16S rRNA* displays greater structural complexity than *12S rRNA*, consistent with functional constraints in mitochondrial ribosome assembly and protein synthesis. We found that its conserved region aligns with those of typical frog species, but there are several unique base variants in the variable region (e.g., A→G transitions at specific sites). These variants could influence the stability of rRNA or relate to mitochondrial translation efficiency. Due to limited rRNA secondary structure data for *Scutiger*, the findings of this study may serve as a valuable reference for molecular evolutionary research on the genus. The taxonomic or adaptive significance of these structural features can be better understood in the future by increasing sample size and conducting comparative analyses of closely related species.

In the mitochondrial genome of *S. ningshanensis*, our analysis revealed a 1480 bp non-coding control region (*D-loop*), the largest intergenic spacer positioned between *tRNA-Pro* and *tRNA-Phe* (Table 1). This conserved element, crucial for mitochondrial DNA replication and transcription initiation, displayed distinct nucleotide characteristics: elevated A + T content (68.45%) coupled with negative GC-skew (−0.349) and AT-skew (−0.058) values. Comparative analysis within Megophryidae species showed conserved region lengths but significant intraspecific variation attributable to differential repeat unit organization. The predominance of dimeric over trimeric repetitive elements, with varying copy numbers, explains the length polymorphisms observed in this functionally essential domain.

The mitochondrial genome of *S. ningshanensis* contains a 1480 bp *D-loop* control region located between tRNA-Pro and tRNA-Phe, characterized by high A + T content (68.45%) and distinct compositional skews (GC-skew: −0.349; AT-skew: −0.058). Comparative genomic analysis across Megophryidae species (*Scutiger*, *Oreolalax*, and *Leptobrachium*) revealed that while this region maintains conserved functional domains essential for replication and transcription initiation, it exhibits substantial length polymorphism attributable to variations in repetitive element organization, including differences in unit length and copy number. Although conserved regulatory motifs have been proposed to reside within these variable regions [36], their exact molecular architecture and functional mechanisms require further investigation to fully understand their role in mitogenome evolution and regulation.

### 3.4. Phylogenetic Analysis

**Figure 7:**
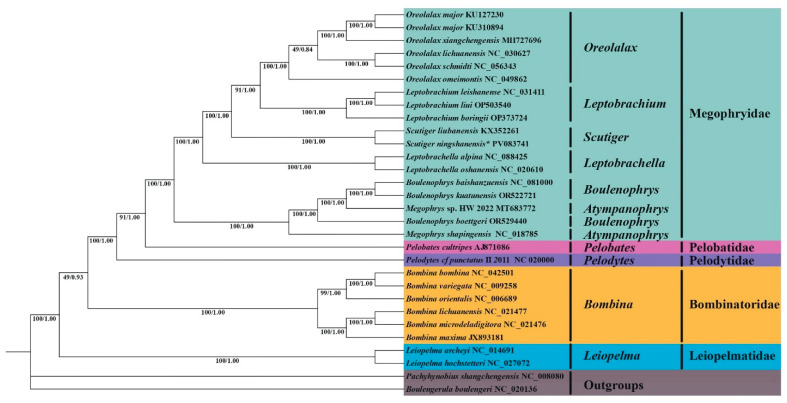
Phylogenetic relationships within Megophryidae derived from ML method based on 13 PCGs; the numbers on the nodes represent the support values of these nodes, the first number represents the bootstrap value of the ML tree, and the second number represents the posterior probability from the BI tree. Species grouped under the same color on the right side of the tree belong to the same taxonomic unit. The species *S. ningshanensis* used in this study is indicated by the Latin name with an asterisk.

Our phylogenetic reconstruction, based on mitochondrial genomes from 30 species of the Megophryidae with those of four other families (Pelobatidae, Pelodytidae, Leiopelmatidae, and Bombinatoridae), in which two species were considered outgroups, yielded concordant topologies using both ML and BI methods (Figure 7). The analysis robustly supported (bootstrap/posterior probability > 0.95) the monophyly of six Megophryidae genera, with *Oreolalax* and *Leptobrachium* forming a distinct clade that established a sister-group relationship with *Scutiger*. Phylogenetic analysis showed that *S. ningshanensis* and *S. liubanensis* are sister species with a close evolutionary relationship. Together with *O. schmidti*, *O. omeimontis*, *O. major*, *O. xiangchengensis*, *O. lichuanensis*, *L. liui*, *L. boringii*, and *L. leishanense*, they form a well-supported clade. Notably, *S. ningshanensis* and *S. liubanensis* occupies a basal position within this clade, suggesting it represents a relatively ancestral lineage among these species. The genera *Atympanophrys* and *Boulenophrys* (including *B. baishanzuensis*, *B. kuatunensis*, and *B. boettgeri*) were recovered as sister taxa within a well-supported branch. External relationships showed Pelobatidae and Pelodytidae as distinct lineages, while Bombinatoridae formed a monophyletic cluster with *Bombina lichuanensis* and *B. microdeladigitora* as sister species. These results provide definitive molecular evidence for: (1) the monophyletic status of major Megophryidae genera, (2) previously unresolved sister-group relationships, and (3) the family’s distinctiveness from related anuran lineages.

### 3.5. Divergence Time of the Megophryidae Species

**Figure 8:**
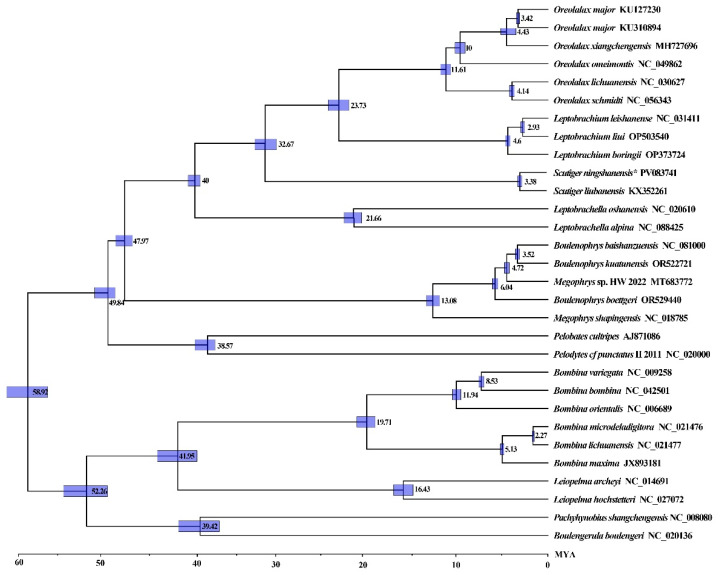
The divergence time of Megophryidae with 95% highest posterior probability density. Numbers nearby nodes refer to divergence times. The “*” represents the current study.

Phylogenetic analyses of Megophryidae using ML and BI methods yielded congruent topologies, consistent with previous studies. Molecular dating estimates place the family’s most recent common ancestor at~47.97 MYA, with subsequent diversification of 16 extant lineages occurring between 3.38–21.66 MYA during the early Miocene (Figure 8). The genera *Leptobrachella*, *Scutiger*, *Leptobrachium*, and *Oreolalax* diverged~40.00 MYA (95% HPD: 39.12–41.35 MYA) in the mid-Oligocene, while *S. ningshanensis* speciated from its closest relatives ~32.67 MYA (95% HPD: 20.74–33.53 MYA) during the late Oligocene. Subsequent radiations occurred in the late Miocene, with *Oreolalax* diversifying 4.14–11.61 MYA and *L. boringii* diverging ~4.60 MYA (95% HPD: 4.52–4.82 MYA). *S. ningshanensis* and *S. liubanensis* separated in 3.38 MYA (95% HPD: 2.91–3.62 MYA). These results indicate that major cladogenic events in Megophryidae occurred primarily during the Oligocene to Miocene transition, with some lineages persisting into the Paleogene.

### 3.6. Selective Pressure Analysis

**Figure 9:**
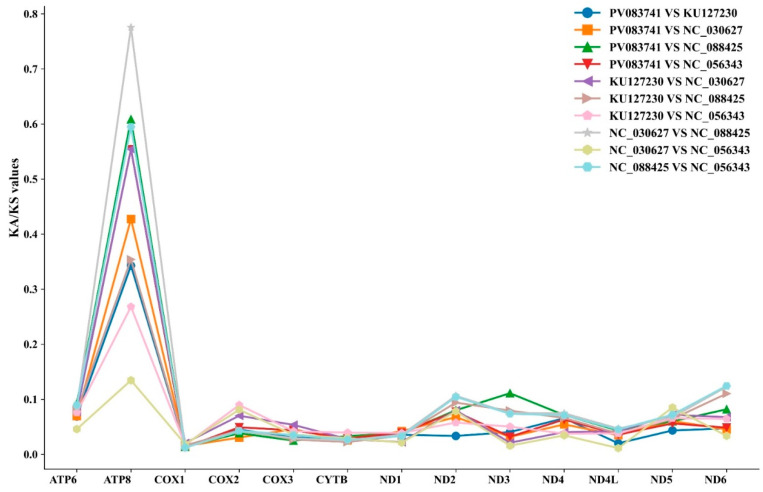
The Ka/Ks values among the *S. ningshanensis* species. Note: PV083741, KU127230, NC_030627, NC_088425, and NC_056343 represent *S. ningshanensis* (this study), *O. major*, *O. lichuanensis*, *L. alpina*, and *O. schmidti*, respectively.

The Ka/Ks ratio (ω) was employed to evaluate selective pressures acting on mitochondrial protein-coding genes (PCGs), with ω > 1 indicating positive selection, ω = 1 representing neutral evolution, and 0 < ω < 1 signifying purifying selection [37]. Our analysis of all 13 PCGs in *S. ningshanensis* revealed ω-values significantly below 1 (Figure 9), demonstrating that purifying selection has dominated their evolutionary trajectory. This evolutionary conservatism supports the utility of these genes for phylogenetic reconstruction within the species. Notably, selective pressure intensity varied substantially among genes: *ATP8* exhibited the highest evolutionary rate (ω = 0.78), while *COX1* showed exceptional conservation with the lowest ω-value (ω = 0.01). These findings indicate that *COX1* undergoes the strongest purifying selection in the *S. ningshanensis* mitogenome, whereas *ATP8* experiences relatively relaxed selective constraints compared to other mitochondrial genes.

## 4. Discussion

Our analysis of the *S. ningshanensis* mitochondrial genome reveals several evolutionarily significant features that warrant discussion. The observed genomic architecture, while conforming to the typical vertebrate mitochondrial blueprint [38,39], presents intriguing characteristics when examined in the broader context of Megophryidae evolution. The conserved gene arrangement, particularly the stable~*ATP8*-*ATP6* overlap region [40,41], suggests strong functional constraints maintaining this organization across the family. The pronounced AT-rich composition and consistent AT skew align with patterns reported in related amphibian lineages [16], potentially reflecting shared evolutionary pressures on mitochondrial DNA structure and function.

Of particular interest is the presence of non-canonical initiation codons in several PCGs, a phenomenon previously noted in other anuran species [42,43]. While such variations might initially appear as random mutations, their persistence across multiple lineages implies potential adaptive significance or relaxed selection pressures in mitochondrial translation initiation. These codon usage patterns, combined with the overall structural conservation, suggest that while the fundamental mitochondrial architecture remains stable in Megophryidae, certain elements may evolve under different selective regimes, possibly reflecting lineage-specific adaptations or neutral evolutionary processes. This study contributes to our understanding of mitochondrial evolution in amphibians by demonstrating how both conservation and variation coexist in the mitogenomic architecture of *S. ningshanensis*. The findings underscore the importance of considering both structural conservation and subtle sequence-level variations when interpreting mitochondrial evolution in phylogenetic contexts.

The evolutionary dynamics of mitochondrial protein-coding genes (PCGs) in *S. ningshanensis* reveal fascinating patterns of molecular adaptation within the Megophryidae family. Our Ka/Ks analysis, a powerful metric for detecting selective pressures [44,45], demonstrates that purifying selection overwhelmingly dominates mitochondrial evolution in these amphibians, as evidenced by consistently low ratios across all PCGs (Figure 9). This pattern strongly suggests that most mitochondrial functions are under stringent evolutionary constraints, likely due to their fundamental role in cellular energy production.

However, the observed variation in evolutionary rates among genes presents a more nuanced evolutionary story. While *COX1* and *Cytb* exhibit extraordinary conservation (Ka/Ks < 0.04)—consistent with their central role in electron transport—the relatively elevated substitution rates in *ATP8*, *ATP6*, and *ND2* (Ka/Ks = 0.10−0.77) may reflect either relaxed structural constraints or positive selection for specific functional adaptations. This differential evolutionary tempo across the mitochondrial genome potentially represents an evolutionary compromise: while core respiratory machinery remains tightly conserved to maintain essential bioenergetic functions, certain components may possess greater evolutionary flexibility to accommodate ecological specialization. These findings carry important implications for understanding high-elevation adaptation in amphibians. The maintenance of strong purifying selection across all PCGs, despite the family’s diversification across varied montane habitats, underscores the remarkable evolutionary stability of mitochondrial energy metabolism systems. This conservation may represent a fundamental constraint on evolutionary innovation in mitochondrial genomes, even in lineages facing challenging environmental conditions.

Our phylogenetic reconstruction, based on comprehensive mitochondrial genome analysis (13 PCGs) of 30 species, provides crucial resolution to longstanding uncertainties surrounding *S. ningshanensis* systematics. The robust congruence between BI and ML topologies (supported by high nodal values) offers compelling evidence for the species’ placement within a well-defined clade containing *Oreolalax*, *Leptobrachium*, and *Leptobrachella*. These findings carry significant implications for understanding Megophryidae evolution, particularly regarding the proposed division of subfamily Megophryinae into 10 genera [45,46].

The observed phylogenetic patterns reveal both consistencies and complexities in Megophryidae systematics. While confirming the monophyly of several genera, our results highlight persistent challenges in delineating evolutionary relationships, particularly within traditionally recognized groups like the *Megophrys* complex. The polyphyletic nature of these assemblages reinforces the growing consensus that molecular systematics must be integrated with morphological data to achieve robust taxonomic classifications [47,48]. This is especially pertinent given the family’s apparent rapid radiation pattern, a phenomenon increasingly recognized as characteristic of Asian amphibian diversification.

Notably, our study identifies several areas requiring further investigation. The unresolved relationships between certain genera (e.g., *Pelodytes* vs. *Pelobates*) and the need for broader taxonomic sampling suggest that current systematic frameworks may not fully capture the family’s evolutionary complexity. Future research directions should prioritize: (1) expanded taxonomic representation, particularly of understudied lineages, (2) incorporation of genomic-scale datasets to complement mitochondrial evidence, and (3) development of integrative approaches that reconcile molecular and morphological data. Such efforts will be essential for constructing a comprehensive evolutionary history of Megophryidae and resolving remaining systematic inconsistencies.

Our phylogenomic reconstruction, incorporating 28 complete mitogenomes, provides compelling evidence for the deep evolutionary history of *Scutiger* and its divergence from *Oreolalax* during the Paleogene (~32.76 MYA). This temporal framework reveals striking correlations between major cladogenic events and Cenozoic climate transitions. The initial Oligocene divergence coincides precisely with global cooling and Antarctic glaciation events, while subsequent radiations during the Neogene mirror the dramatic temperature fluctuations of the Miocene Climatic Optimum and subsequent Pliocene cooling.

The temporal concordance between Megophryidae diversification and global climate reorganization strongly supports climate-mediated speciation mechanisms [49]. Particularly noteworthy is the accelerated lineage splitting during periods of extreme thermal variability, suggesting that these amphibians may be particularly sensitive to temperature-driven selection pressures. This pattern holds important implications for understanding current responses to anthropogenic climate change.

From a conservation perspective, our results establish *S. ningshanensis* as an evolutionarily distinct lineage that persisted through multiple climate regimes. This long-term evolutionary persistence, coupled with its montane-endemic distribution, underscores its significance as a conservation priority. The mitochondrial genomic framework developed here not only clarifies phylogenetic relationships but also establishes an essential baseline for future studies of amphibian adaptation to environmental change and the development of targeted conservation strategies for ancient high-elevation lineages.

## 5. Conclusions

This study presents the complete mitochondrial genome (17,282 bp) of *S. ningshanensis*, revealing conserved vertebrate architecture with characteristic AT-rich codon bias and purifying selection across all PCGs. Structural analysis identified typical cloverleaf tRNAs, except for the DHU-deficient *trnS1*. Phylogenetic reconstruction robustly supports the monophyly of *Oreolalax*, *Leptobrachium*, and *Leptobrachella*, with *Oreolalax*-*Leptobrachium* forming a sister clade to *Scutiger*. The topology confirms current taxonomy, showing *Boulenophys* and *Atympanophys* as sister genera, while revealing complex genus-level diversification within Megophryidae. Molecular dating suggests *S. ningshanensis* originated ~32.76 MYA during accelerated Paleogene diversification. Strong purifying selection (Ka/Ks < 1) was observed, particularly in respiratory genes (*COX1-3*, *ND1/4L/6*), indicating functional constraints during high-elevation adaptation. These results provide: (1) a genomic foundation for Megophryidae systematics, (2) evidence for selective pressures shaping mitochondrial evolution, and (3) a temporal framework for understanding the family’s radiation in Asia.

## Figures and Tables

**Table 1:** PCR primers for the *S. ningshanensis* mitochondrial genome.

No.	Primer Name	Sequence 5′-3′	Primer Length (bp)	References
1	CTF1	ATTAAGATAAAGCCCTTCTAGAA	23	This study
	CTR1	AATACCATTGGTGTCCCACG	20	
2	CTF2	CAAGAYGCRRYHTCHCCNATYATAGAAGA	29	[20]
	CTR2	CCTTCWCGRAYNAYRTCTCGYCAYCAYTG	29	
3	CTF3	GCMCACCAAGCWCAYGCHTWYCAYATRGT	29	[20]
	CTR3	GADCCDGCRATDGGDGCYTCDACRTG	26	
4	CTF4	CACTACGCAGCAGACACCTC	20	This study
	CTR4	CAAGGGAAGGTCCTATCAAGT	21	
5	CTF5	ATGGTGGTATAATAGTATGGTGT	23	This study
	CTR5	GTTGTTGGGAATAAGGGTGT	20	
6	CTF6	ACCTCATACGCAAACTCAGC	20	This study
	CTR6	TACCATCATTTTAATAGGTGGA	22	
7	CTF7	CCCACATGTATAATTAACAGATT	23	This study
	CTR7	GGAGTAATCTTTCGTTTTGTAT	22	
8	CTF8	TTCGCAAAGCAAATACCCACA	21	This study
	CTR8	CGCCGACTAATATCAATTTG	20	
9	CTF9	CCACACCYHCAAGGGHAYTCAGCAGT	26	[20]
	CTR9	CTTYGCACGGTYAGRRTACCGCGGCCGT	28	
10	CTF10	CCCGCCTGTTTACCAAAAACAT	22	[20]
	CTR10	ACRTTRAANCCNGANACHAGTTCWGAYTC	29	
11	CTF11	CGRGCHGTHGCHCAAACNATYTCHTAYGA	29	[20]
	CTR11	AAGCTCKCTGGAWWGAGYGTTTAGCTGTTAA	31	
12	CTF12	GTCGCCCAAACAATCTCATATGA	23	[20]
	CTR12	AGGAGGGCTTTATCTTAAT	19	

**Table 2:** Annotation of the mitochondrial genome of *S. ningshanensis*.

Gene	Direction	Position		Size (bp)	IGS (bp)	Codon	
		From	To			Start	Stop
*tRNA-Phe*	H	2	66	65	0		
*12S rRNA*	H	67	1001	935	−4		
*tRNA-Val*	H	998	1067	68	1		
*16S rRNA*	H	1069	2680	1610	−3		
*tRNA-Leu*	H	2678	2752	75	0		
*ND1*	H	2753	3730	978	154	ATG	TAA
*tRNA-Ile*	H	3885	3995	71	87		
*tRNA-Gln*	L	4083	4153	71	7		
*tRNA-Met*	H	4161	4229	68	0		
*ND2*	H	4230	5273	1044	0	ATG	TAA
*tRNA-Trp*	H	5274	5342	69	4		
*tRNA-Ala*	L	5347	5416	70	0		
*tRNA-Asn*	L	5417	5489	73	−1		
*rep_origin*	H	5489	5520	32	−3		
*tRNA-Cys*	L	5518	5580	63	0		
*tRNA-Tyr*	L	5581	5650	70	1		
*COX1*	H	5652	7203	1552	1	GTG	T−−
*tRNA-Ser*	L	7205	7275	71	4		
*tRNA-Asp*	H	7280	7347	68	1		
*COX2*	H	7349	8036	688	0	ATG	T−−
*tRNA-Lys*	H	8037	8110	74	0		
*ATP8*	H	8111	8290	180	−10	GTG	TAA
*ATP6*	H	8281	8962	682	0	ATG	T−−
*COX3*	H	8963	9746	784	0	ATG	T−−
*tRNA-Gly*	H	9747	9816	70	0		
*ND3*	H	9817	10,159	343	0	ATG	T−−
*tRNA-Arg*	H	10,160	10,228	69	0		
*ND4L*	H	10,229	10,525	297	−7	ATG	TAA
*ND4*	H	10,519	11,896	1378	0	ATG	T−−
*tRNA-His*	H	11,897	11,965	69	0		
*tRNA-Ser*	H	11,966	12,032	67	0		
*tRNA-Leu*	H	12,033	12,103	71	0		
*ND5*	H	12,104	13,924	1821	15	ATG	TAG
*ND6*	L	13,940	14,449	510	0	ATG	AGG
*tRNA-Glu*	L	14,450	14,518	69	2		
*CYTB*	H	14,521	15,661	1141	0	ATG	T−−
*tRNA-Thr*	H	15,662	15,731	70	2		
*tRNA-Pro*	L	15,734	15,802	69	0		
*D-loop*	H	15,803	17,282	1480	0		

**Table 3:** Nucleotide composition and skewness values of *S. ningshanensis* mitogenome.

Gens	Size(bp)	A%	T%	G%	C%	A + T%	G + C%	AT-Skew	GC-Skew
*Mitogenome*	17,282	28.54	32.51	14.3	24.64	61.05	38.94	−0.065	−0.265
*PCGs*	11,398	26	34.87	14.67	24.46	60.87	39.13	−0.145	−0.250
*PCGs(1st)*	3797	27.44	27.23	23.12	22.2	54.67	45.33	0.0039	0.0203
*PCGs(2nd)*	3797	17.99	42.09	12.96	26.97	60.07	39.93	−0.4011	−0.3509
*PCGs(3rd)*	3797	32.6	35.19	7.95	24.26	67.79	32.21	−0.0381	−0.5061
*tRNAs*	1504	29.79	29.26	20.88	20.08	59.05	40.96	0.009	0.0195
*rRNAs*	2547	32.08	27.21	17.71	23.01	59.29	40.71	0.082	−0.13
*D-loop*	1480	32.23	36.22	10.27	21.28	68.45	31.55	−0.058	−0.349
*ND1*	978	26.07	33.33	26.28	14.31	59.4	40.59	−0.122	−0.294
*ND2*	1044	25.96	37.36	25.48	11.21	63.32	36.69	−0.18	−0.389
*COX1*	1552	25.97	33.31	23.71	17.01	59.28	40.72	−0.123	−0.164
*COX2*	688	29.36	31.1	24.85	14.68	60.46	39.53	−0.028	−0.257
*ATP8*	180	29.44	38.89	23.33	8.33	68.33	31.67	−0.138	−0.473
*ATP6*	682	25.51	36.36	26.39	11.73	61.87	38.12	−0.175	−0.384
*COX3*	784	23.72	33.42	25.89	16.96	62.1	42.86	−0.169	−0.208
*ND3*	343	23.32	38.78	23.62	14.29	62.1	37.9	−0.248	−0.246
*ND4L*	297	24.92	33	28.96	13.13	57.92	42.09	−0.139	−0.376
*ND4*	1378	27.07	34.83	25.33	12.77	61.9	38.1	−0.125	−0.329
*ND5*	1821	27.4	35.15	24.66	12.8	62.55	37.45	−0.123	−0.316
*ND6*	510	18.82	38.43	11.18	31.57	57.25	42.75	−0.342	0.477
*CYTB*	1141	26.03	35.14	24.45	14.37	61.17	38.83	−0.149	−0.259

## Data Availability

Mitochondrial genome sequence data supporting the findings of this study are openly available from GenBank of the National Center for Biotechnology Information (NCBI) at https://www.ncbi.nlm.nih.gov (accession number: PV083741), accessed on 30 July 2025.
